# Supplementary material for: Partial Protection of Goats against Haemonchus contortus Achieved with ADP-Ribosylation Factor 1 Encapsulated in PLGA Nanoparticles
Source: Vaccines (Basel). 2024 Oct 18;12(10):1188. doi: 10.3390/vaccines12101188 (PMC11511444; doi:10.3390/vaccines12101188)
Supplement: Supplementary file 1 [file vaccines-12-01188-s001.zip › vaccines-3207558-supplementary.pdf]

vaccines

Supplementary Figure S1

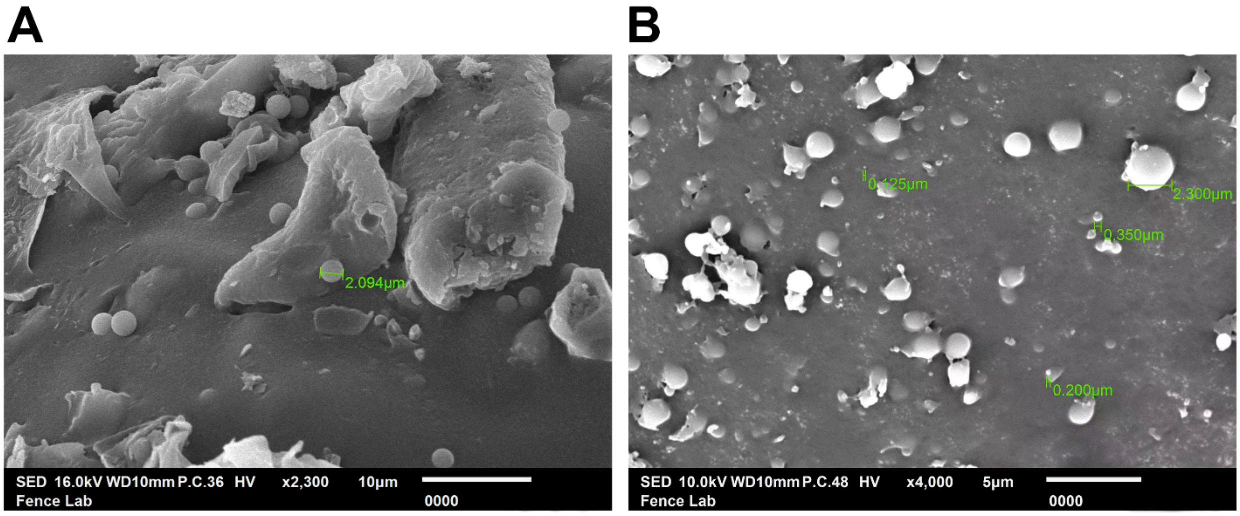

An investigation was conducted to determine the optimal concentration of polyvinyl alcohol (PVA) for a study involving the design of two distinct PVA concentration gradients. The morphology of the resultant poly (lactic-co-glycolic acid) nanoparticles (PLGA NPs) was assessed through scanning electron microscopy. (A) 1% PVA (B) 4% PVA. Scale bars: (A = 10 μm, B = 5 μm).

Supplementary Figure S2

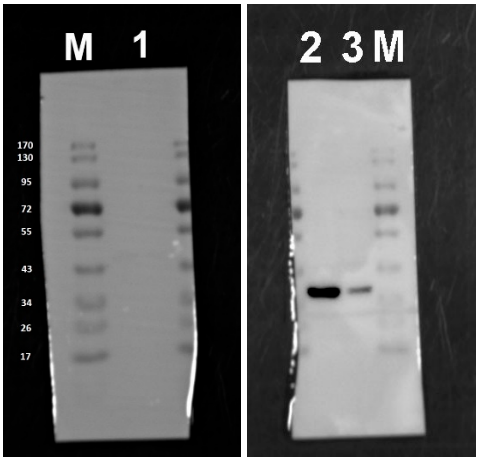

The western blot analysis confirmed the presence of the purified recombinant HcARF1 (rHcARF1) protein by specifically detecting it with rat anti-rHcARF1 sera. Lane M shows the molecular weight marker for protein size reference. Lane 1, which was probed with normal rat sera, serves as a negative control to ensure probing specificity. Lanes 2 and 3 display different concentrations of total excretory-secretory (ES) proteins from *H. contortus*, probed with antibodies derived from Sprague-Dawley (SD) rats immunized with rHcARF1, demonstrating the recognition of rHcARF1 within the ES protein sample.

Table S1. Densitometry readings/intensity ratio (Band size) measured by ImageJ software 1.51.

Line . No	Area	Mean	Min	Max
2	0.003	186.486	73	255
3	0.001	104.913	57	169
